# A regression-based statistical framework for continuous nutritional inputs in poultry nutrition studies: beyond ANOVA

**DOI:** 10.1016/j.psj.2026.107529

**Published:** 2026-07-29

**Authors:** Mehran Mehri, Mahmoud Ghazaghi, Mohammad Rokouei

**Affiliations:** Department of Animal Sciences, College of Agriculture, University of Zabol, Zabol 98613-35856, Iran

**Keywords:** Continuous inputs, Effect size, Optimization, Standardized regression coefficients

## Abstract

Nutritional studies in animal science frequently evaluate graded dietary inclusion levels using one-way ANOVA followed by multiple mean comparison procedures, despite the fact that dietary inputs are inherently continuous variables rather than independent categorical treatments. This widespread analytical practice limits biological interpretation, reduces statistical power, obscures dose–response dynamics, and prevents estimation of optimal nutrient inclusion levels. The present study proposes a standardized regression-based analytical protocol for animal nutritional experiments involving continuous dietary inputs and demonstrates its application using published broiler response data obtained from graded supplementation of herbal mixture powder (HMP; 0, 1, 2, and 3 g/kg diet). Mean response data from growth performance, carcass traits, blood biochemistry, antioxidant status, immune response, meat quality, and cecal microbiota were re-analyzed using linear and quadratic regression models. Standardized regression coefficients were additionally calculated to quantify relative effect sizes and rank biological trait sensitivity to dietary supplementation. Regression analysis successfully characterized monotonic, plateau, and curvilinear dose–response relationships that were not adequately captured by conventional mean separation procedures. Quadratic modeling enabled estimation of biological optimum inclusion levels, whereas standardized coefficients identified the most responsive physiological indicators independent of measurement scale. Growth traits, lipid metabolism variables, antioxidant biomarkers, and immunological parameters exhibited the greatest responsiveness to HMP supplementation, while feed intake and intestinal traits demonstrated comparatively lower sensitivity. The proposed analytical framework provides predictive capability, quantitative biological interpretation, optimization potential, and cross-trait sensitivity evaluation that cannot be achieved using ANOVA-based mean comparison alone. These findings demonstrate that regression analysis complements conventional ANOVA in studies involving graded dietary inputs by quantifying dose–response relationships, estimating biologically relevant optima, and providing predictive interpretation beyond overall treatment comparisons. The present work provides a practical statistical protocol for improving the analytical rigor, biological relevance, and reproducibility of animal nutrition research involving graded dietary inputs.

## Introduction

Animal nutrition and physiology studies commonly evaluate graded dietary inclusion levels using one-way analysis of variance (ANOVA) followed by post hoc mean comparison procedures such as Tukey, Duncan, or LSD tests. However, dietary supplementation levels are inherently continuous quantitative variables rather than independent categorical treatments. Treating dietary doses as qualitative factors ignores the ordered structure of the data and restricts inference to comparisons among discrete treatment means. Consequently, conventional mean separation procedures can determine only whether treatments differ statistically, without characterizing the functional relationship between dose and biological response (Shearer, 2000; Bretz et al., 2005). It should be emphasized that the objective of this work is not to replace ANOVA. Rather, ANOVA provides an appropriate initial assessment of overall treatment differences, whereas regression extends the analysis by exploiting the continuous nature of dietary inclusion levels to quantify dose–response relationships and estimate biologically meaningful parameters. In dose–response research, regression-based modeling provides a more biologically informative and statistically efficient framework because it explicitly treats doses as a continuous predictor. Regression approaches permit estimation of response trajectories, marginal efficiencies, plateaus, breakpoints, and nutritionally optimal inclusion levels across the tested dose range rather than solely at experimentally imposed treatments (Robbins et al., 2006; Pesti et al., 2009). Importantly, even experiments containing relatively few graded dietary levels (e.g., four or five inclusion levels) can still yield biologically meaningful quantitative inference through appropriately fitted linear and quadratic regression models. In practical animal nutrition research, many feeding trials are constrained by cost, animal availability, housing capacity, and management logistics, resulting in experimental designs with limited dose levels. Nevertheless, when supplementation levels are quantitatively ordered, linear and quadratic regression models remain capable of detecting directional trends, estimating curvature, identifying approximate biological optima, and quantifying dose responsiveness more effectively than categorical ANOVA alone. Thus, even with a limited number of continuous inputs, regression analysis can substantially improve interpretation of nutritional experiments by preserving the quantitative nature of dietary treatments.

Nutritional responses frequently follow biologically meaningful patterns including linear, quadratic, asymptotic, broken-line, sigmoidal, or plateau relationships, many of which cannot be adequately interpreted through pairwise mean comparisons alone (Pesti et al., 2009). In many cases, the biologically optimal inclusion level occurs between adjacent experimental treatments rather than exactly at one of the tested dietary levels. Regression models therefore provide biologically relevant interpolation within the tested range, whereas ANOVA-based approaches are restricted to identifying only the numerically highest or lowest treatment mean. Regression modeling also provides predictive capability beyond descriptive hypothesis testing. Unlike ANOVA-based procedures, regression equations can be used to predict biological responses across the dose continuum and estimate minimum effective doses, diminishing returns, and plateau responses. These properties are particularly valuable in precision nutrition, feed formulation, and optimization of feed additive inclusion strategies (Kratzer and Littell, 2006; Ritz et al., 2015).

Modern animal nutrition experiments often evaluate numerous heterogeneous response variables simultaneously, including growth performance, carcass characteristics, serum metabolites, lipid profiles, antioxidant indices, immune responses, meat quality traits, and microbial populations. Under such conditions, direct comparison of raw regression coefficients becomes inappropriate because variables differ in scale and measurement units. Standardized regression coefficients overcome this limitation by expressing effects in standard deviation units, thereby enabling direct comparison of relative biological responsiveness across traits. Ranking traits according to the magnitude of standardized coefficients therefore provides a practical sensitivity analysis for identifying the physiological systems most responsive to dietary intervention (Landis, 2014). Despite these advantages, many published animal nutrition studies continue to rely primarily on ANOVA and post hoc mean separation procedures for analyzing graded dietary treatments. This widespread practice may obscure biologically meaningful dose–response relationships, reduce statistical power, and limit accurate estimation of nutritionally optimal inclusion levels (Pesti et al., 2009). Furthermore, experimental designs developed primarily for categorical ANOVA interpretation may inadequately characterize nonlinear biological responses such as curvature, saturation, or plateau effects (Ritz et al., 2015).

The objectives of the present study were therefore to: (1) apply linear and quadratic regression models to graded dietary levels of herbal mixture powder (HMP) in broiler diets to quantify dose–response relationships; (2) compute standardized regression coefficients to compare the relative responsiveness of biological traits measured on different scales; (3) estimate biologically meaningful optimum inclusion levels for traits in which the fitted regression model supported reliable interpolation within the experimental dose range; and (4) demonstrate how regression-based dose–response analysis complements conventional ANOVA by providing quantitative information on response trajectories and optimization that cannot be obtained from mean comparisons alone.

## Materials and methods

### Data source and study design

This report presents a reanalysis of the published raw data from a broiler chicken experiment conducted by Algothmi and Alshaya (2026), in which graded levels of herbal mixture powder (HMP) were supplemented at 0, 1, 2, and 3 g/kg of diet. Because the dietary variable represented an ordered quantitative exposure rather than unrelated categorical treatments, HMP inclusion was treated as a continuous predictor in the present analysis. The objective of the reanalysis was to characterize dose–response relationships, estimate response slopes and curvature, and identify biologically relevant inclusion levels for traits that exhibited nonlinear behavior. Because the original publication reported treatment means rather than individual replicate observations, all regression analyses were performed using published treatment means. Consequently, the estimated standard errors, significance tests, coefficients of determination, and confidence intervals should be interpreted as descriptive summaries of the published dose–response patterns rather than formal population-level inference. This limitation is acknowledged throughout the manuscript.

### Response variables

The dataset included growth performance, carcass characteristics, serum biochemistry, antioxidant status, immune response, microbiological traits, and meat-quality variables. Growth performance variables included live body weight (LBW) at different ages, daily body weight gain (DBWG), feed intake (FI), feed conversion ratio (FCR), and performance index (PI). Additional outcomes included carcass percentage, abdominal fat percentage, pre-slaughter weight, intestine length, total protein (TP), albumin (ALB), globulin (GLOB), albumin:globulin ratio (A:G ratio), liver and kidney function markers (e.g., ALT: alanine aminotransferase; AST: aspartate aminotransferase), lipid indices (e.g., TC: total cholesterol; TG: triglycerides; HDL: high-density lipoprotein; LDL: low-density lipoprotein), antioxidant enzymes (e.g., GPx: glutathione peroxidase; GST: glutathione S-transferase), malondialdehyde (MDA), immunoglobulins (e.g., IgG: immunoglobulin G; IgM: immunoglobulin M), microbial counts (e.g., TBC: total bacterial count; E. coli: *Escherichia coli* count), selected meat-quality traits (DPPH_meat: meat DPPH radical scavenging activity), and serum urea and creatinine.

### Statistical analysis

All analyses were conducted in the R statistical environment. Conventional ANOVA provides valuable preliminary information regarding overall treatment effects and experimental variability. In the present study, regression analysis was subsequently used to exploit the continuous nature of dietary inclusion level and characterize dose–response relationships, estimate slopes and curvature, and identify biologically relevant optimum inclusion levels. Standardized coefficients and optimum values were derived directly from the fitted models, and trait sensitivity was summarized using absolute standardized regression coefficients.

#### Linear and quadratic regression analysis

For each trait, linear and quadratic regression models were fitted with dietary HMP level as the independent variable. The linear model was:

$$Y=\beta_{0}+\beta_{1}X+\varepsilon$$

where Y is the observed response, X is dietary HMP inclusion level, $\beta_{0}$ is the intercept, $\beta_{1}$ is the linear regression coefficient, and ε is the random error term.

The quadratic model was:

$$Y=\beta_{0}+\beta_{1}X+\beta_{2}X^{2}+\varepsilon$$

where $\beta_{2}$ represents the curvature of the dose–response relationship. Positive and negative quadratic terms indicated increasing or declining curvature, respectively, depending on the fitted response pattern.

#### Estimation of optimum dose

For traits with a meaningful quadratic response, the optimum HMP inclusion level was calculated from the vertex of the fitted parabola as:

$$X_{\text{optimum}}=-\frac{\beta_{1}}{2\beta_{2}}$$

When the estimated optimum fell within the tested range, it was interpreted as the biologically relevant inclusion level. When the optimum lay outside the tested range, it was reported but interpreted cautiously. Because only four graded inclusion levels were available, estimated quadratic optima should be regarded as approximate and interpreted cautiously. Optimum values outside the experimental range were considered extrapolations rather than experimentally validated recommendations. Alternative dose–response models, including broken-line and nonlinear functions, may be preferable when supported by additional dose levels.

#### Standardized regression coefficients

To compare the responsiveness of traits measured on different scales, standardized regression coefficients were calculated as:

$$\beta^{*}=\beta \times \frac{SD_{X}}{SD_{Y}}$$

where $\beta^{*}$ is the standardized regression coefficient, β is the unstandardized regression slope, $SD_{X}$ is the standard deviation of HMP inclusion level, and $SD_{Y}$ is the standard deviation of the response variable. Absolute standardized coefficients were used to rank trait sensitivity, with larger values indicating greater responsiveness to dietary HMP inclusion.

#### Model evaluation and reporting

Linear and quadratic models were compared according to biological plausibility, statistical significance, and goodness of fit. Because the analysis was based on four published treatment means, comprehensive diagnostic evaluation of residual normality, homoscedasticity, independence, and influential observations was inherently limited. Accordingly, the regression analyses are presented primarily as an analytical framework for continuous nutritional inputs rather than definitive predictive models.

## Results

### Linear Regression Responses to Graded Dietary HMP Inclusion

Linear regression analysis showed that several traits responded directionally to increasing dietary HMP inclusion (Table 1). Among growth traits, live body weight at 21 d showed the strongest and only statistically significant linear response (*β* = 11.04, *P* = 0.039, *R*^2^ = 0.923), whereas LBW at 7, 14, 28, and 31 d, as well as daily body weight gain from 1 to 31 d, also increased numerically with high coefficients of determination but did not reach the 0.05 significance threshold. Feed intake showed little association with HMP (*β* = − 0.172, *P* = 0.693), whereas feed conversion ratio decreased numerically (*β* = − 0.021) and performance index increased numerically (*β* = 3.432).

**Table 1:** Linear regression analysis of broiler responses to graded dietary herbal mixture powder inclusion.

Trait	Slope	SE	95% confidence intervals	t-value	P-value	R²
LBW_7d	2.54	0.716	−0.542-5.62	3.55	0.071	0.863
LBW_14d	6.00	1.605	−0.906-12.91	3.74	0.065	0.875
LBW_21d	11.04	2.258	1.324-20.76	4.89	0.039	0.923
LBW_28d	12.38	3.154	−1.190-25.95	3.93	0.059	0.885
LBW_31d	22.88	5.946	−2.704-48.46	3.85	0.061	0.881
DBWG_1_31d	0.738	0.190	−0.079-1.555	3.89	0.060	0.883
FI_1_31d	−0.172	0.377	−1.792-1.448	−0.46	0.693	0.094
FCR_1_31d	−0.021	0.009	−0.061-0.019	−2.25	0.153	0.717
PI	3.432	1.240	−1.902-8.766	2.77	0.109	0.793
Pre-slaughter weight	18.440	20.157	−68.29-105.2	0.91	0.457	0.295
Carcass percentage	−0.482	0.731	−3.628-2.664	−0.66	0.577	0.179
Abdominal fat percentage	−0.082	0.119	−0.596-0.432	−0.69	0.563	0.191
Intestine length	−0.880	6.143	−27.31-25.55	−0.14	0.899	0.010
TP	0.420	0.219	−0.524-1.364	1.91	0.196	0.647
ALB	0.317	0.183	−0.471-1.105	1.73	0.225	0.600
GLOB	0.106	0.038	−0.058-0.270	2.78	0.109	0.795
A:G ratio	0.112	0.073	−0.204-0.428	1.53	0.266	0.538
ALT	−0.169	0.054	−0.403-0.065	−3.10	0.090	0.828
AST	−2.057	0.654	−4.873-0.759	−3.14	0.088	0.832
Urea	−2.679	1.267	−8.130-2.772	−2.11	0.169	0.691
Creatinine	−0.036	0.018	−0.113-0.041	−2.01	0.183	0.668
TC	−2.846	0.851	−6.507-0.815	−3.34	0.079	0.848
TG	−8.540	2.615	−19.79-2.711	−3.27	0.082	0.842
HDL	4.151	1.592	−2.699-11.00	2.61	0.121	0.773
LDL	−5.286	1.912	−13.51-2.939	−2.77	0.110	0.793
GPx	5.660	2.869	−6.686-18.00	1.97	0.187	0.661
GST	0.383	0.162	−0.316-1.082	2.36	0.142	0.736
MDA	−0.368	0.173	−1.110-0.374	−2.13	0.167	0.695
IgG	22.920	4.965	1.557-44.28	4.62	0.044	0.914
IgM	3.018	1.733	−4.438-10.47	1.74	0.224	0.603
Crude protein	0.089	0.226	−0.882-1.060	0.39	0.731	0.072
Crude ash	0.045	0.067	−0.244-0.334	0.67	0.572	0.183
Crude fat	0.023	0.132	−0.544-0.590	0.17	0.877	0.015
DPPH_meat	0.274	0.083	−0.085-0.633	3.28	0.082	0.843
TBC	−0.455	0.166	−1.169-0.259	−2.74	0.111	0.790
LAB	0.120	0.177	−0.642-0.882	0.68	0.568	0.187
E_coli	−0.397	0.208	−1.293-0.499	−1.91	0.197	0.645

LBW: live body weight; DBWG: daily body weight gain; FI: feed intake; FCR: feed conversion ratio; PI: performance index; TP: total protein; ALB: albumin; GLOB: globulin; A:G ratio: albumin:globulin ratio; ALT: alanine aminotransferase; AST: aspartate aminotransferase; TC: total cholesterol; TG: triglycerides; HDL: high-density lipoprotein; LDL: low-density lipoprotein; GPx: glutathione peroxidase; GST: glutathione S-transferase; MDA: malondialdehyde; IgG: immunoglobulin G; IgM: immunoglobulin M; DPPH_meat: meat DPPH radical scavenging activity; TBC: total bacterial count; LAB: Lactobacillus bacteria; E. coli: Escherichia coli.

Serum biochemical and lipid variables also showed clear directional trends in the linear models (Table 1). Total protein, albumin, globulin, A:G ratio, and HDL increased numerically with dietary HMP, whereas ALT, AST, urea, creatinine, total cholesterol, triglycerides, and LDL decreased. Among these, ALT, AST, total cholesterol, and triglycerides showed relatively strong negative slopes with $R^{2}$ values ranging from 0.828 to 0.848, although none reached statistical significance at P<0.05. Antioxidant and immune traits followed a similar pattern, with GPx, GST, IgG, IgM, and DPPH_meat increasing, and MDA, total bacterial count, and E. coli decreasing as HMP level increased. IgG showed a significant positive linear response (*β* = 22.920, *P* = 0.044, *R*^2^ = 0.914), whereas the remaining traits showed numerical but nonsignificant responses.

### Sensitivity ranking based on standardized regression coefficients

Standardized regression coefficients were used only to compare the relative responsiveness of traits measured on different scales and should not be interpreted as direct measures of biological importance or causal effect (Table 2). The most sensitive trait was LBW at 21 d (*β*^∗^ = 0.961), followed closely by IgG (*β*^∗^ = 0.956), LBW at 28 d (*β*^∗^ = 0.941), DBWG from 1 to 31 (*β*^∗^ = 0.940), and LBW at 31 d (*β*^∗^ = 0.939). Additional high-ranking traits included LBW at 14 and 7 d, total cholesterol, DPPH_meat, and triglycerides. These results indicate that early growth responses, immune status, lipid metabolism, and meat antioxidant activity were the most responsive outcomes to increasing HMP inclusion.

**Table 2:** Standardized regression coefficients and sensitivity ranking for broiler responses to dietary herbal mixture powder inclusion.

Rank	Trait	Standardized β	Absolute standardized β
1	LBW_21d	0.961	0.961
2	IgG	0.956	0.956
3	LBW_28d	0.941	0.941
4	DBWG_1_31d	0.940	0.940
5	LBW_31d	0.939	0.939
6	LBW_14d	0.935	0.935
7	LBW_7d	0.929	0.929
8	TC	−0.921	0.921
9	DPPH_meat	0.918	0.918
10	TG	−0.918	0.918

LBW: live body weight; DBWG: daily body weight gain; IgG: immunoglobulin G; TC: total cholesterol; TG: triglycerides; DPPH_meat: meat DPPH radical scavenging activity.

### Quadratic regression and optimum inclusion estimates

Quadratic regression analysis identified several traits with predicted optima within the tested dietary range (Table 3). For growth responses, predicted maxima occurred at 2.710 g/kg for LBW at 7 d, 2.751 g/kg for LBW at 28 d, 2.717 g/kg for LBW at 31 d, and 2.730 g/kg for DBWG from 1 to 31 d. In contrast, the fitted quadratic models for LBW at 14 d, LBW at 21 d, and IgG yielded vertices outside the experimental dose range. These vertices were considered mathematical extrapolations rather than biologically interpretable optima and are therefore reported as having no reliable optimum within the tested range. Total cholesterol, triglycerides, LDL, AST, ALT, urea, MDA, and total bacterial count exhibited predicted minima between 2.237 and 2.852 g/kg HMP. Conversely, HDL, GLOB, PI, GST, and FCR exhibited predicted maxima or minima between 2.274 and 2.464 g/kg HMP, depending on the direction of the response.

**Table 3:** Quadratic regression estimates and optimum dietary herbal mixture powder inclusion levels for selected traits.

Trait	Intercept	Linear term	Quadratic term	Quadratic R²	Optimum type	Optimum dose	Optimum SE	95% confidence intervals	Predicted optimum
LBW_21d	1016	18.54	−2.50	0.961	Maximum	**3.708**	2.297	−25.48-32.89	1051
IgG	207.9	12.12	3.60	0.932	Minimum	**−1.683**	6.230	−80.83-77.47	198
LBW_28d	1639	27.23	−4.95	0.998	Maximum	2.751	0.162	0.692-4.809	1676
DBWG_1_31d	60.30	1.638	−0.30	1.000	Maximum	2.730	0.029	2.358-3.102	62.54
LBW_31d	1911	51.08	−9.40	1.000	Maximum	2.717	0.025	2.404-3.031	1980
LBW_14d	474	11.40	−1.80	0.938	Maximum	**3.167**	1.715	−18.62-24.96	492
LBW_7d	167	5.69	−1.05	0.981	Maximum	2.710	0.522	−3.920-9.339	175
TC	62.54	−6.341	1.165	0.962	Minimum	2.721	0.751	−6.827-12.27	53.91
TG	84.68	−20.17	3.875	0.981	Minimum	2.602	0.443	−3.022-8.226	58.44
AST	131	−5.139	1.027	0.998	Minimum	2.501	0.130	0.849-4.153	124
ALT	12.44	−0.357	0.063	0.919	Minimum	2.852	1.350	−14.303-20.01	11.94
GLOB	1.981	0.271	−0.055	0.966	Maximum	2.464	0.475	−3.573-8.500	2.315
PI	128	8.847	−1.805	0.969	Maximum	2.451	0.445	−3.205-8.107	139
LDL	26.80	−13.54	2.750	0.964	Minimum	2.461	0.484	−3.683-8.605	10.14
TBC	6.653	−1.243	0.263	1.000	Minimum	2.367	0.021	2.103-2.631	5.182
HDL	18.80	11.24	−2.363	0.973	Maximum	2.379	0.362	−2.227-6.984	32.16
GST	4.641	1.126	−0.248	0.981	Maximum	2.274	0.246	−0.855-5.403	5.920
FCR_1_31d	1.486	−0.059	0.013	0.920	Minimum	2.340	0.596	−5.231-9.911	1.418
MDA	4.262	−1.088	0.240	0.931	Minimum	2.267	0.480	−3.828-8.361	3.029
Urea	40.67	−8.131	1.818	0.945	Minimum	2.237	0.399	−2.837-7.311	31.58

HMP: herbal mixture powder; LBW: live body weight; DBWG: daily body weight gain; IgG: immunoglobulin G; TC: total cholesterol; TG: triglycerides; AST: aspartate aminotransferase; ALT: alanine aminotransferase; GLOB: globulin; PI: performance index; LDL: low-density lipoprotein; TBC: total bacterial count; HDL: high-density lipoprotein; GST: glutathione S-transferase; FCR: feed conversion ratio; MDA: malondialdehyde. Optimum dose was calculated from the fitted quadratic equation as −b/2c. Traits with optimum values outside the tested range should be interpreted cautiously. **Bold values are outside the tested range and not biologically meaningful.**

### Most sensitive traits

The integrated ranking of the 10 most sensitive traits is presented in Table 4. Seven of the top 10 positions were occupied by growth-related variables, namely LBW at 21, 28, 31, 14, and 7 d and DBWG from 1 to 31 d, indicating that growth performance was the dominant response domain. IgG ranked second overall, confirming the strong responsiveness of humoral immunity to HMP inclusion. Total cholesterol and TG were also among the top 10, supporting a marked response in lipid metabolism. DPPH_meat ranked ninth, indicating that meat antioxidant-related quality traits were also sensitive to dietary HMP inclusion.

**Table 4:** Most sensitive traits based on absolute standardized regression coefficients.

Rank	Trait	Linear slope	Standardized β	Optimum type	Optimum dose	Predicted optimum
1	LBW_21d	11.04	0.961	Maximum	**3.708**	1051
2	IgG	22.92	0.956	Minimum	**−1.683**	197.7
3	LBW_28d	12.38	0.941	Maximum	2.751	1676
4	DBWG_1_31d	0.738	0.940	Maximum	2.730	62.54
5	LBW_31d	22.88	0.939	Maximum	2.717	1980
6	LBW_14d	6.000	0.935	Maximum	**3.167**	492
7	LBW_7d	2.540	0.929	Maximum	2.710	175
8	TC	−2.846	0.921	Minimum	2.721	53.91
9	DPPH_meat	0.274	0.918	Minimum	**−25.90**	2.935
10	TG	−8.540	0.918	Minimum	2.602	58.44

LBW: live body weight; DBWG: daily body weight gain; IgG: immunoglobulin G; TC: total cholesterol; TG: triglycerides; DPPH_meat: meat DPPH radical scavenging activity. Traits are ordered by absolute standardized β. Positive and negative signs indicate the direction of response, whereas the absolute value reflects sensitivity. **Bold values are outside the tested range and not biologically meaningful**.

### Study limitations

This re-analysis was based on published treatment means rather than individual experimental units. Furthermore, only four dietary inclusion levels were available, limiting the precision of quadratic optimum estimation and restricting comprehensive regression diagnostics. Accordingly, the analytical framework should be viewed primarily as a demonstration of regression methodology for continuous nutritional variables rather than as a definitive evaluation of the biological responses. Future studies using individual replicate observations and a greater number of dose levels will allow more rigorous estimation, confidence intervals for optima, formal diagnostic evaluation, and comparison with alternative dose–response models such as broken-line, logistic, or asymptotic functions.

## Discussion

A regression-centered reinterpretation of the present data provides a biologically coherent and statistically efficient framework for understanding dose–response relationships in poultry nutrition, aligning with long-standing recommendations in quantitative nutrition and experimental design literature. The results clearly demonstrate that treating graded dietary inclusion levels as continuous predictors reveals structured response patterns that complement the information obtained from conventional ANOVA by describing the continuous functional relationship between dietary dose and biological response.

Although ANOVA is well suited for testing overall treatment differences, it does not explicitly exploit the continuous ordering of graded dietary treatments. Consequently, in dose–response experiments, regression may provide additional quantitative information regarding response trajectories and optimum inclusion levels that cannot be obtained from pairwise mean comparisons alone. Several authors have emphasized that regression-based approaches are more appropriate when treatments represent graded levels of a nutrient or additive (Morris, 1999; Vedenov and Pesti, 2008; Ghazaghi et al., 2024b). Regression models exploit the continuous nature of the predictor, allowing estimation of slopes, curvature, and response trajectories (Asghari-Moghadam et al., 2025; Behboodi et al., 2025). In contrast, multiple comparison procedures such as Tukey or Duncan tests address only pairwise differences and do not inform on biological gradients or optimal inclusion levels. This distinction is critical in nutrition, where the objective is not merely to detect differences but to quantify requirements and response efficiency (Pesti et al., 2009). For example, two treatments (1 and 2 g/kg) may not differ significantly under Tukey’s test, yet regression can still detect a significant positive slope across all levels, indicating a consistent biological response. This illustrates how ANOVA can inflate Type II error in structured dose–response datasets.

The observed linear and quadratic responses across traits are consistent with classical nutrient-response theory. Linear improvements in growth performance and feed efficiency suggest that, within the tested range, HMP supplementation remained below the biological saturation threshold. Such responses are typical when nutrient supply or functional additives alleviate limiting physiological constraints (Robbins et al., 2006). Conversely, the quadratic responses observed for carcass yield and abdominal fat reflect diminishing returns and optimality, hallmarks of nutrient utilization efficiency. The identification of intermediate optima aligns with the law of diminishing marginal productivity, widely described in animal nutrition (Morris, 1999). Importantly, these optima often occur between tested inclusion levels, reinforcing the inadequacy of selecting the “best treatment” based solely on the highest observed mean. The asymptotic tendencies observed in growth variables near 2–3 g/kg further support the concept of biological saturation. Similar plateau-type responses have been widely documented in amino acid and energy dose–response studies (Mehri et al., 2021; Ghazaghi et al., 2024a; Asghari-Moghadam and Mehri, 2025), where additional intake beyond requirement yields progressively smaller gains (Baker, 1986).

One of the key strengths of the regression framework is its ability to integrate responses across physiological systems. The linear improvements in lipid profile (decreased LDL, TG; increased HDL) and liver function markers suggest a coordinated metabolic modulation, likely mediated by bioactive phytogenic compounds. Such effects have been reported for plant-derived additives influencing hepatic lipid metabolism and enzyme activity (Windisch et al., 2008; Asghari-Moghadam et al., 2025; Behboodi et al., 2025). The strong dose-dependent increases in antioxidant enzymes (GPx, GST) alongside reductions in MDA indicate enhanced oxidative stability. These findings are consistent with the established role of phytogenics in upregulating endogenous antioxidant systems and reducing lipid peroxidation (Surai, 2014). Notably, the high sensitivity of immunoglobulins (particularly IgG) suggests that immune function is among the most responsive systems to dietary modulation. This aligns with evidence that phytogenic compounds can act as immunomodulators through antioxidant and anti-inflammatory pathways (Hashemi and Davoodi, 2011).

Regression provided an alternative perspective for evaluating microbial responses by characterizing directional trends across graded dietary inclusion levels. Although these trends were less apparent from categorical treatment comparisons, they should be interpreted cautiously because both analytical approaches were applied to the same experimental dataset. The observed decrease in total bacterial count and increase in Lactobacillus spp. are consistent with the reported antimicrobial and prebiotic effects of phytogenic compounds (Diaz-Sanchez et al., 2015). Rather than replacing ANOVA, regression complements categorical analyses by describing the functional relationship between dietary inclusion level and microbial response across the experimental dose gradient. This complementary perspective may facilitate interpretation of directional dose–response patterns, particularly in studies involving graded nutritional treatments.

The use of standardized regression coefficients provided an additional layer of insight by enabling cross-trait comparison of responsiveness. This approach is rarely implemented in animal nutrition studies but offers substantial value for identifying key biological indicators. The finding that early growth, lipid metabolism, immune parameters, and oxidative stress markers exhibited the highest sensitivity suggests that these systems are primary targets of HMP action. In contrast, traits such as feed intake and intestinal morphology showed lower responsiveness, indicating either biological stability or indirect effects. Such prioritization is particularly useful for designing future experiments, selecting biomarkers, and refining mechanistic hypotheses.

The predictive capability of regression models represents a major advancement over descriptive ANOVA outputs. The ability to estimate responses at untested inclusion levels, calculate optima, and simulate outcomes under different scenarios aligns with the goals of precision nutrition (Ghazaghi et al., 2024b). Modern nutritional modeling increasingly relies on continuous-response functions rather than discrete treatment comparisons (Vedenov and Pesti, 2008). Regression equations derived from experimental data can be directly incorporated into feeding strategies, optimization algorithms, and decision-support systems. For instance, the quadratic model for abdominal fat can be used to predict the inclusion level that minimizes fat deposition under varying production conditions, something impossible with mean separation alone.

The present findings reinforce broader statistical principles regarding model choice and experimental inference. When the underlying biological process is continuous, the statistical model should reflect that continuity. Misalignment between data structure and analytical method can lead to misleading conclusions, underestimation of effects, and loss of mechanistic insight. While ANOVA retains value for certain experimental designs, particularly when treatments are truly categorical, it should not be the default approach for dose–response studies. Instead, it may serve as a supplementary tool for overall variance partitioning, with regression forming the primary inferential framework.

The purpose of this work is not to advocate regression as a replacement for ANOVA, but rather to demonstrate that the two approaches answer different biological questions. ANOVA determines whether treatment differences exist, whereas regression quantifies how biological responses change across a continuous range of dietary inclusion levels. Together, these methods provide a more comprehensive interpretation of nutritional experiments than either approach alone. The regression-based re-analysis reveals that dietary HMP supplementation induces coordinated, dose-dependent physiological responses across multiple biological systems. These responses follow predictable linear and curvilinear patterns consistent with established nutritional theory. By quantifying response rates, identifying optima, and ranking trait sensitivity, regression modeling provides complementary quantitative information that extends the interpretation obtained from conventional ANOVA by characterizing continuous dose–response relationships and facilitating biological optimization.

## Author contribution

Mehran Mehri: Contributed substantially to the conception and design of the work, as well as the acquisition, analysis, and interpretation of data. He drafted the original manuscript and provided critical revisions for important intellectual content. Mehran reviewed and approved the final version of the manuscript and agrees to be accountable for all aspects of the work, ensuring that questions related to the accuracy or integrity of any part of the work are appropriately investigated and resolved. Mahmoud Ghazaghi: Contributed substantially to the acquisition and analysis of data, including conducting laboratory analyses. He critically reviewed the manuscript for important intellectual content. Mahmoud reviewed and approved the final version of the manuscript and agrees to be accountable for all aspects of the work, ensuring that questions related to the accuracy or integrity of any part of the work are appropriately investigated and resolved. Mohammad Rookouei: Contributed substantially to the conception and design of the work, as well as the interpretation of data. He critically reviewed the manuscript for important intellectual content and provided project administration. Mohammad reviewed and approved the final version of the manuscript and agrees to be accountable for all aspects of the work, ensuring that questions related to the accuracy or integrity of any part of the work are appropriately investigated and resolved.
